# Distinct and enhanced hygienic responses of a leaf‐cutting ant toward repeated fungi exposures

**DOI:** 10.1002/ece3.9112

**Published:** 2022-07-17

**Authors:** Aryel C. Goes, Pepijn W. Kooij, Laurence Culot, Odair C. Bueno, Andre Rodrigues

**Affiliations:** ^1^ Department of General and Applied Biology São Paulo State University (UNESP) Rio Claro Brazil; ^2^ Department of Biodiversity São Paulo State University (UNESP) Rio Claro Brazil

**Keywords:** *Atta sexdens*, disease, pathogens, secondary exposures, social immunity

## Abstract

Leaf‐cutting ants and their fungal crops are a textbook example of a long‐term obligatory mutualism. Many microbes continuously enter their nest containing the fungal cultivars, destabilizing the symbiosis and, in some cases, outcompeting the mutualistic partners. Preferably, the ant workers should distinguish between different microorganisms to respond according to their threat level and recurrence in the colony. To address these assumptions, we investigated how workers of *Atta sexdens* sanitize their fungal crop toward five different fungi commonly isolated from the fungus gardens: *Escovopsis* sp., *Fusarium oxysporum*, *Metarhizium anisopliae*, *Trichoderma spirale*, and *Syncephalastrum* sp. Also, to investigate the plasticity of these responses toward recurrences of these fungi, we exposed the colonies with each fungus three times fourteen days apart. As expected, intensities in sanitization differed according to the fungal species. Ants significantly groom their fungal crop more toward *F. oxysporum*, *M. anisopliae*, and *Syncephalastrum* sp. than toward *Escovopsis* sp. and *T. spirale*. Weeding, self‐, and allogrooming were observed in less frequency than fungus grooming in all cases. Moreover, we detected a significant increase in the overall responses after repeated exposures for each fungus, except for *Escovopsis* sp. Our results indicate that *A. sexdens* workers are able to distinguish between different fungi and apply distinct responses to remove these from the fungus gardens. Our findings also suggest that successive exposures to the same antagonist increase hygiene, indicating plasticity of ant colonies' defenses to previously encountered pathogens.

## Graphical abstract

The leaf‐cutting ant *Atta sexdens* discriminates between different antagonistic fungi and apply distinct responses to remove them from the fungus garden. Also, successive exposures to the same antagonist increase sanitization in the fungus garden.

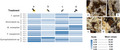

## 1 INTRODUCTION

**FIGURE 1:**
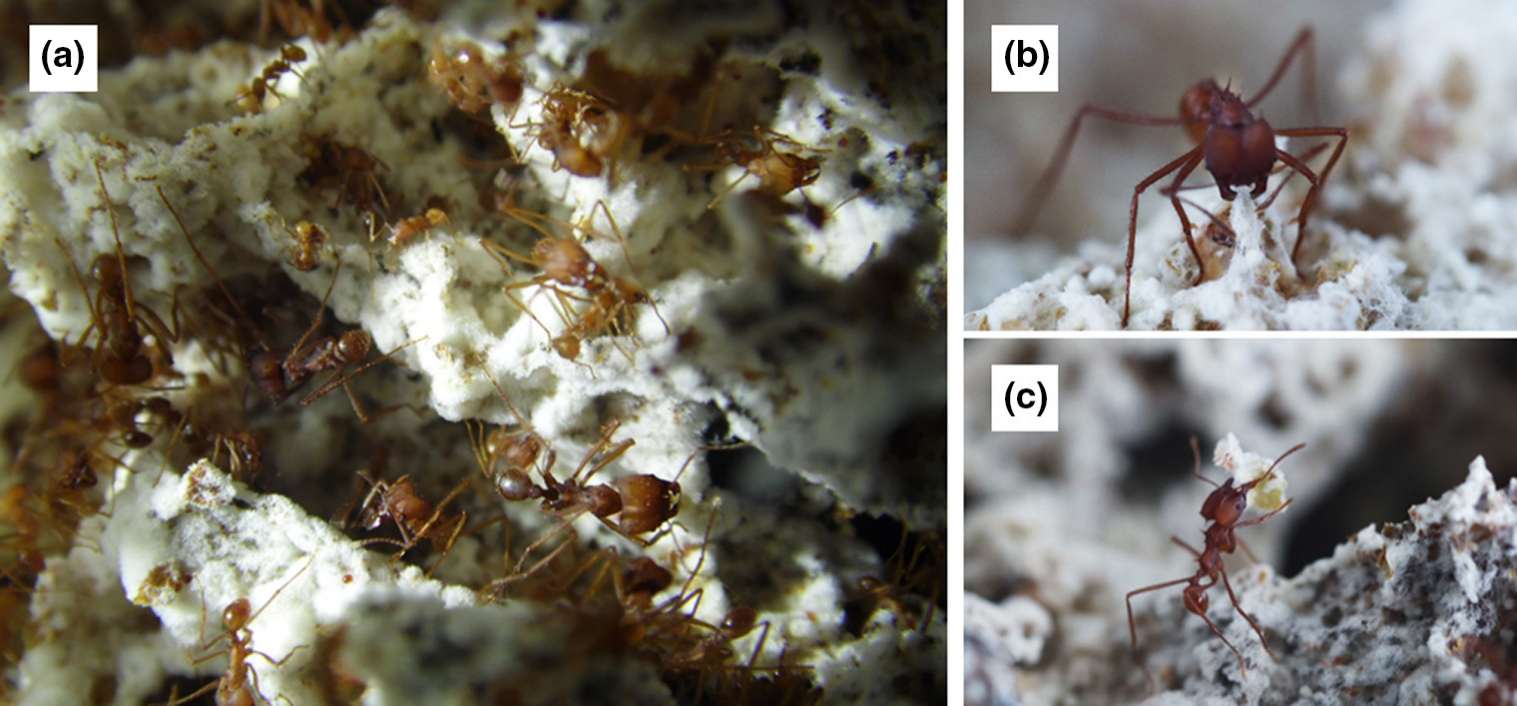
The leaf‐cutting ant *Atta sexdens* maintains an obligatory mutualism with the basidiomycete fungus *Leucoagaricus gongylophorus*. (a) ants cultivate the fungus in a fungus garden. (b and c) workers apply several sanitary strategies to reduce resource exploration by antagonistic microorganisms. One of these strategies, fungus weeding (b and c), is the removal of a contaminated piece of the fungus garden by a single worker and later discarded at the dump chamber. Photograph “a” by Aryel C. Goes, and photographs “b and c” by Quimi Vidaurre Montoya

Associations with microorganisms provide new functional and ecological features for organisms throughout evolution (Baldauf, 2003; Caldera et al., 2009; McFall‐Ngai et al., 2013; Moran, 2006). To maintain these benefits, hosts must ensure screening and selecting specific symbionts during environmental acquisition or transmission to offspring (Biedermann & Kaltenpoth, 2014). They also need to detect and recognize antagonistic microbes in advance to prevent their uptake and avoid costly interactions (Biedermann & Kaltenpoth, 2014; Sachs et al., 2004), as seen in many fungus‐growing insects (Davis et al., 2013; Huler et al., 2011). In leaf‐cutting ants (Hymenoptera: Formicidae: Myrmicinae: Attini; subtribe: Attina), studies demonstrated the wide array of hygienic defenses toward unwelcome microbes that exploit the same resources and niches as their fungal crops (Bonadies et al., 2019; Currie et al., 1999; Currie & Stuart, 2001; Fernández‐Marín et al., 2009; Rocha et al., 2014; Yek et al., 2012) (Figure 1). The survival of their fungal cultivars (*Basidiomycota: Agaricales*, including *Leucoagaricus gongylophorus*) and the microbiota found in the colony (Aylward et al., 2014; Barcoto et al., 2020; Khadempour et al., 2020; Suen et al., 2010) relies on a combination of the ants' ability to defend themselves and their partners using cleaning strategies (Goes et al., 2020).

Generally, leaf‐cutting ants apply secretions containing antimicrobial compounds from their metapleural glands directly onto the fungus garden (Fernández‐Marín et al., 2006, 2015; Nilssøn‐Moller et al., 2018; Yek et al., 2012) or even nestmates (Little et al., 2006). In addition, some attine ant species use antibiotics produced by mutualistic Actinobacteria (e.g., *Pseudonocardia*) to complement their chemical barriers (Currie et al., 1999; Li et al., 2018). Leaf‐cutting ant behaviors such as self‐ and allogrooming, that is, the removal of contaminants from itself or another individual, respectively (Morelos‐Juárez et al., 2010; Richard & Errard, 2009), preparation and cleaning of plant material used as a substrate for the fungus crop (Mangone & Currie, 2007; Quinlan & Cherrett, 1977), and the removal of foreign conidia or infected pieces of the fungus garden (grooming and weeding, respectively, Currie & Stuart, 2001; Nilssøn‐Moller et al., 2018), are prophylactic and suppressive strategies against invasions from various pathogens. Spatial avoidance (Cremer et al., 2007; Stroeymeyt et al., 2014) and division of labor (Farji‐Brener et al., 2016; Hart & Ratnieks, 2001; Waddington & Hughes, 2010) also reduce the likelihood of infection by decreasing contact between infected and healthy workers.

Several alien fungi with distinct lifestyles were reported in *Atta* and *Acromyrmex* colonies, as generalist mycopathogens (e.g., *Trichoderma* and *Syncephalastrum*), entomopathogens (e.g., *Beauveria* and *Metarhizium*, Rodrigues et al., Rodrigues & Pagnocca, 2005, Rodrigues et al., 2008; Goffré & Folgarait, 2018), and mycoparasites (i.e., *Escovopsis*, Currie et al., 2003). In theory, if leaf‐cutting ants recognize the diversity of alien fungi entering their colonies, defensive responses are expected to follow their threat levels (Goes et al., 2020; Mighell & Van Bael, 2016). For instance, increased hygienic responses toward *Escovopsis* when compared with *Trichoderma viride* corroborate a possible distinction between mycelium and/or conidia of a specialist and a generalist fungal pathogen, respectively (Currie & Stuart, 2001). Likewise, stronger responses were observed to the obligate entomopathogen *Metarhizium anisopliae* than to the facultative entomopathogen *Aspergillus flavus* in different contexts, that is, food, environment, and nestmates (Tranter et al., 2015). Even physical removal of fungi appears to be species‐specific, as strains of the genera *Escovopsis* (Christopher et al., 2021a), *Trichoderma*, and *Xylaria* (Mighell & Van Bael, 2016) are removed in higher rates than others. Although it suggests the ability of some attine ants to discriminate and respond to alien fungi, such mechanisms remain uncertain in many *Atta* and *Acromyrmex* species. Also, how these ants react to repeated encounters to the same fungi, that is, secondary infections (Walker & Hughes, 2009), is yet to be explored.

Considering the multiple and recurrent threats in their system that could imbalance the symbiosis, thus it is reasonable to ask whether the cleaning responses of leaf‐cutting ants evolved to be plastic. In this scenario, we hypothesize that if hygienic strategies applied vary depending on the diversity of fungi, then distinct fungi must trigger distinct responses in *Atta sexdens* colonies. Furthermore, if these ants have plasticity against repeated infections to the same strains, we expect increased sanitization in secondary encounters with a previously inoculated pathogen (homologous exposure). Alternatively, if the system relies on generalist strategies, we expect a similar pattern in responses regardless the fungus and no increase of sanitation in following encounters. To investigate this, we applied bioassays to measure the sanitization of *A. sexdens* toward four different pathogenic fungi (i.e., *Escovopsis* sp., *M. anisopliae*, *Syncephalastrum* sp., and *Trichoderma spirale*), and one nonpathogenic fungus (i.e., *Fusarium oxysporum*). We analyzed how these ant colonies: (1) respond to the fungal species selected for this study, and (2) whether they increase hygienic responses in secondary exposures to each of these microorganisms.

## 2 MATERIALS AND METHODS

### 2.1 Collection and maintenance of colonies

We collected 38 *A. sexdens* colonies of similar age and size, that is, fungus garden volume and a random number of workers, pupae, larvae, and eggs. Colonies came from newly mated queens collected shortly after the mating flight in October–November 2016 at Itirapina Ecological Station, São Paulo, Brazil (Coordinates: −22.225662 −47.840134). From field‐collected colonies, we randomly chose colonies for the experiments based on subjective criteria (as in Barcoto et al., 2017): (1) the vivacity of the queen by looking at the presence of eggs deposited during two weeks and her active movements, (2) the health of the fungus garden, that is, the absence of contamination and the continuous degradation of plant material, and (3) the activity of the colony in regard to foraging behavior of the ants during two weeks, that is, acceptance, cutting, and disposal of leaves at the fungus garden. Raffles and observations were carried out blindly to avoid bias during treatment designation and data accuracy (Kardish et al., 2015). We maintained the colonies in a glass container (30 × 22 × 4 cm, l × w × h) connected to two plastic pots, one for foraging and one for dump area, kept at 23–24°C, under 12 h:12 h light–dark cycle. We started experiments when the fungus garden had filled the whole glass container to avoid bias in regard to differences in number of workers. To maintain the humidity within colonies, we evenly spread 1 cm of plaster at the bottom of the glass arena. We provided fresh leaves of *Hibiscus* sp., *Mangifera* sp., and oat flakes daily and alternately, when necessary, to control the excess of humidity at the glass chamber. We did not feed the colonies on the day of the experiment to avoid interference with ants' behaviors. Lastly, the dump chambers were cleaned with paper towels once or twice a week, and one hour before the experiments start.

We randomly selected five colonies to receive the treatment, that is, fungal conidia, and five colonies to receive the control, that is, sham solution (0.05% Tween® 80 diluted in water, Sigma‐Aldrich, MO, US). At the end of each experiment, we let the colonies recover and eliminate remnant conidia for one month to reuse them in the next treatments (Figure 1). During this interval, we checked the colonies' health daily to ensure they could be used in further experiments, using the same criteria as in Barcoto et al. (2017). When a queen died during this period, or if the colony showed stress or signs of imminent collapse, we discarded the colony and replaced it with a new one from the pool of collected colonies. A total of 10 colonies had to be discarded and replaced this way during the whole study. In total, we used 20 colonies from the pool of 38 field‐collected colonies. We only discarded colonies in the recovery period between experiments of different fungal species, but never between different exposures. After one month, if considered healthy, the ten colonies previously used were randomized regardless of their previous treatments, that is, fungus exposure or control, followed by a new raffle to nominate which would receive the fungus or sham exposures. Thus, colonies were always randomized before the start of a new set of experiments.

### 2.2 Selection and cultivation of fungi

**FIGURE 2:**
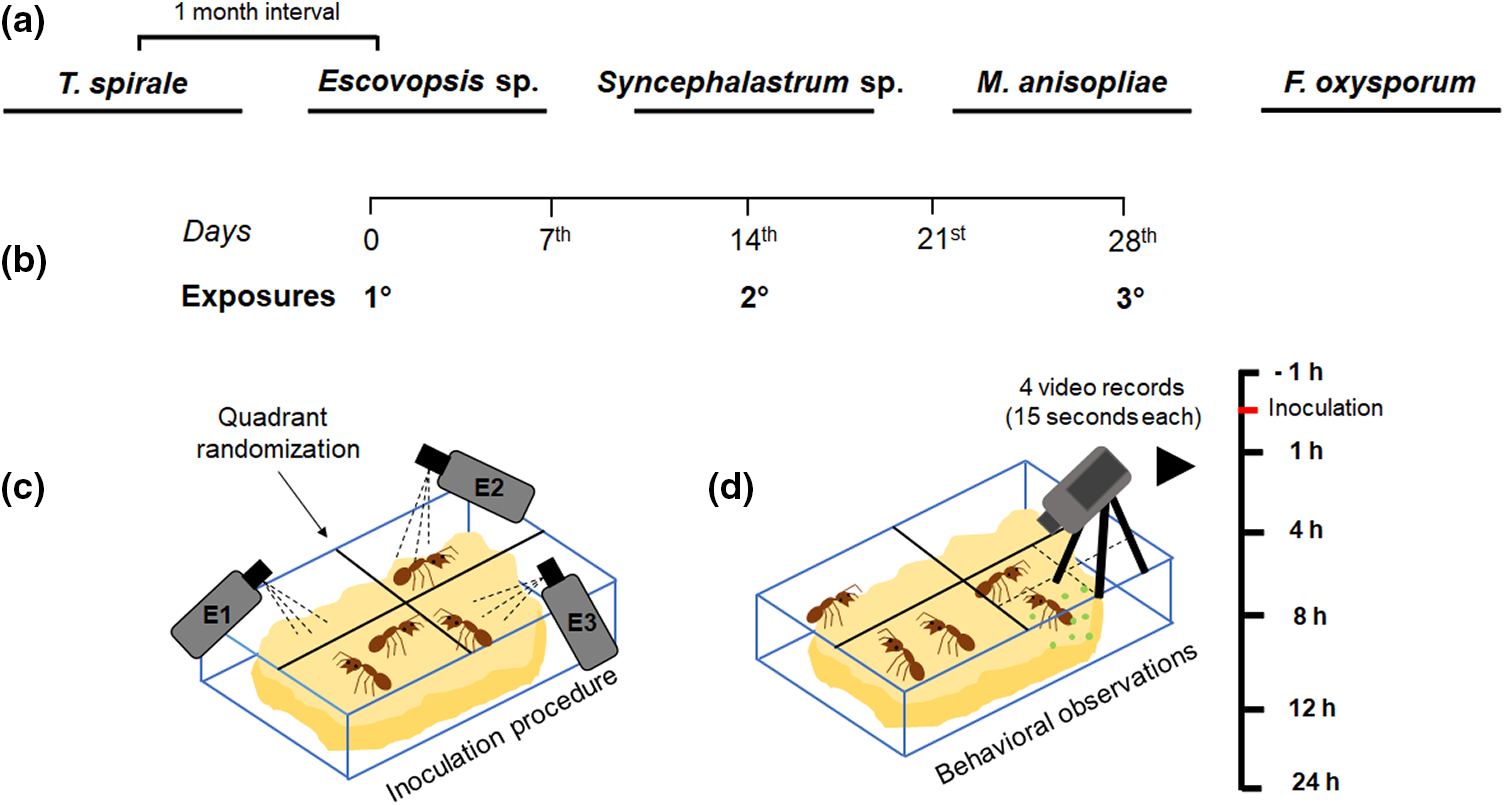
Schematics of the experimental setup. (a) the experiments of the different fungal species, that is, *Escovopsis* sp., *Fusarium oxysporum*, *Metarhizium anisopliae*, *Syncephalastrum* sp., and *Trichoderma spirale*, were carried out with a one‐month interval between the end of the previous and start of the next experiment, using ten colonies of *Atta sexdens* (five treatment and five control). (b) each treatment consisted of three successive exposures (E1, E2, and E3) using live conidia or sham solution as control with a delay of 14 days between each exposure. (c) the inoculation was applied on a previously raffled quadrant of the fungus garden, that is, 3 of 4 quadrants received the treatments once. (d) the treated quadrant was divided into four sectors for behavioral observations and we recorded each sector once per recording session (15 s each). We recorded at 1 h before and at 1, 4, 8, 12, and 24 h post‐inoculation

To explore how ants respond to different fungi, we selected five species that are commonly isolated from leaf‐cutting ant colonies and surrounding areas (Rodrigues et al., 2008; Van Bael et al., 2009) (Figure 2a). Each chosen species has a distinct lifestyle: three species that are potentially harmful to the fungus garden, that is, the antagonistic *Trichoderma spirale* (strain LESF 117, Rodrigues et al., 2008; Rocha et al., 2017), the mycoparasitic *Escovopsis* sp. (strain LESF 021, Currie et al., 2003) and the generalistic pathogen *Syncephalastrum* sp. (strain LESF 127, Barcoto et al., 2017); a common entomopathogen, that is, *Metarhizium anisopliae* (strain LESF 206, Lacerda et al., 2010; Lopez & Orduz, 2003), and a soil‐born fungus unknown to be harmful to the system, that is, *Fusarium oxysporum* (strain LESF 333, Rodrigues et al., 2008). We are aware that most of these species have conidia, but that *Syncephalastrum* sp. has spores (or merospores). For clarity of the text, we used conidia throughout the text.

All five strains mentioned above were previously isolated from *A. sexdens* colonies and cryopreserved as axenic cultures in 10% glycerol at −80°C at the Laboratory of Ecology and Systematics of Fungi (LESF, UNESP, Rio Claro). The fungal strains were revived from cryopreservation by inoculating conidia on Potato Dextrose Agar (20 g L^−1^ of Agar, 4 g L^−1^ of Potato Extract, and 20 g L^−1^ of Dextrose; PDA, Neogen® Culture Media, MI, USA) and incubating the Petri dishes at 25°C for 10 days in an incubator. We checked daily for contamination of the cultures. Once reactivated, the fungi were transferred to slant tubes with 2% Malt Extract Agar (20 g L^−1^ of Malt extract and 15 g L^−1^ of Agar; Neogen® Culture Media, MI, USA) and kept at 8°C as working stocks. Before inoculation of the colonies with each fungus, the respective strains were transferred to PDA and incubated at 25°C for seven days.

### 2.3 Sequential exposures and inoculation procedure

To investigate whether *A. sexdens* colonies increase hygienic responses in sequential exposures to the same fungus, we performed the treatments three times (E1, E2, and E3) with either fungi or sham solution (Figure 2a). The three exposures were applied 14 days apart (Figure 2b). Between the encounters, we checked (1) if the colonies crashed, (2) showed signs of stress (e.g., reduced harvesting and an increase of the fungal cultivar being discarded), or (3) if a queen died. We did not observe clear signs of stress or dead queens between exposures throughout all the experiments. To inoculate different portions of the colony equally, we virtually divided the fungus garden into four quadrants of equal size (15 × 11 cm), delimited by a dashed line on the glass lid of the main arena (Figure 2c). At the start of each experiment, we raffled these quadrants to define which would receive the first, second, and third exposure, with each quadrant only receiving a single treatment. We did the randomization process in all fungus experiments.

We prepared conidia suspensions by collecting mycelium from 7‐ to 10‐days‐old cultures (following Osti & Rodrigues, 2018). We suspended all material in 0.05% Tween^®^ 80 in water, in sterile 10‐ml plastic tubes. To clean the conidial suspensions from any mycelium, we vortexed the material for one minute to mechanically separate them. Next, we filtered the suspensions using a sterile glass pipette with cotton at one end to obtain the clean conidia with Tween^®^ 80 in a new plastic tube. We measured the concentration of the conidial suspensions using a Neubauer chamber and diluted them to 10^6^ conidia per ml. Then, 1 ml of the conidia suspensions was transferred to a sterile 5 ml hand‐sprinkler and equally sprayed on the fungus garden at the randomly raffled quadrants (Figure 1c). To ensure the viability of conidia, we pipetted 20 μl of the suspensions on PDA and spread this on the surface with a Drigalski spatula. We incubated the conidia at 25°C for seven days and checked daily for growth. For the sham solution, we used 0.05% Tween^®^ 80 in water only.

### 2.4 Behavioral observations and sampling

We recorded the colonies using a Sony HDR – CX150/B (3.1 megapixels) video camera by directing it only at the quadrant which received the treatment or control exposure (Figure 1). We assessed the responses of workers 1 h before and at 1, 4, 8, 12, and 24 h after exposure. As the camera could not capture the full quadrant, we recorded the different portions of the fungus garden that received the treatment by changing the camera position (Figure 1d), resulting in four consecutive videos of 15 s each accounting a total of 60 s. We took particular care to avoid disturbing the colonies during camera manipulation, only turning the lights from the room on at the recording (60 s × 6 time intervals [hour −1, 1, 4, 8, 12, and 24] = 6 min per colony × 10 colonies equals approx. 60 min of light exposure per day).

We examined 3600 video records, that is, in total 15 h of material, registering the sanitization behaviors presented by workers with the instantaneous focal sampling method (Martin & Bateson, 2007); each video of 15 s was paused at 5, 10, and 15 s, and the number of workers displaying each behavior at these times was counted using a hand‐counter (VMC–4, Vonder, Brazil). As these snapshot counts are technical replicates, we averaged the behaviors counted in these intervals to obtain a single value for each of the four consecutive videos, that is, the videos from each subquadrant. To reduce observational and counting bias, a single examiner (ACG) watched the videos and registered behaviors. Also, we took care not to count the same individual twice.

We focused on four hygienic behaviors from the repertoire applied by leaf‐cutting ants to protect themselves and the fungus garden (Currie & Stuart, 2001; Nilssøn‐Moller et al., 2018):

1. Worker selfgrooming, when a single ant stops at a portion of the fungus garden and brushes the antennae on the front legs; or when the ant cleans the antennae and the legs by passing them through the mouthparts, removing particles with their glossa.
2. Worker allogrooming, when one or more ants are nearby another ant, i.e., the receiver, which usually remains motionless. The grooming ant(s) lick(s) with its/their mouthparts opened, using the glossa, the receiver ant, moving slightly to cover the main body parts.
3. Fungus grooming, when an ant is immobile at a fixed point of the fungus garden. The antennae remain motionless and parallel, with the ends touching the tips of the mandibles. The ant opens its mandibles and makes small retracting movements with the head, pulling off a tiny portion of the fungal crop and storing it inside the mouthparts (Currie & Stuart, 2001).
4. Fungus weeding, when an ant stops its leg movements and points the antennae toward a specific portion of the fungus garden. The ant uses its mandibles to either cut or detach a large infected piece of the garden, pulling it off and carrying it to discard at the dump chamber (Currie & Stuart, 2001). The disposing of the fungus could not be identified since our video only recorded a specific quadrant. Therefore, we counted fungus weeding only when the removal of a large piece of the garden was identified.

### 2.5 Data analysis

Fungus grooming was the most triggered behavior shown by the ants, approximately 97% of the total hygienic responses (see Results section), while the other behaviors were more rarely expressed, with many zero occurrence in the dataset. Thus, we pooled the number accounted for each behavior, at their specific time interval and exposure, into a single category named “total cleaning responses.” Also, because no treatment was present at one hour before inoculating, the counted behaviors at this point were used only to set the baseline activity of ants before any interference in the system, that is, the number of behaviors at zero hours was subtracted from each of other time points.

To assess if the “total cleaning responses” varied between the fungal treatments, we fitted GLMMs analyses (generalized linear mixed model; Dobson & Barnett, 2008; Winter, 2013) with a negative binomial distribution, based on residual diagnostics for hierarchical regression models (DHARMa package; Hartig, 2020). The total cleaning responses was our response variable in the model and the interaction between treatment and exposure as a fixed effect. As the same colony and fungus garden received several inoculations, we assumed that there was dependency between measures of the different exposures. Thus, we included the exposure as a random factor nested within quadrants and quadrants within colony (Schielzeth & Nakagawa, 2013), because the measured quadrant was always located in the same colony. To check whether a specific hygienic behavior varied in response to the fungal species, we ran additional GLMM analysis, but with each behavior as our response variable.

To test whether each fungal species provoked an increase in hygienic responses compared with the control and between exposures, we applied GLMM analyses for each fungal species. The goal of this intertreatment and exposure comparison was to examine increase in hygienic actions. Here, treatment and the interaction between the treatment and the exposure were taken as fixed effects. The exposure nested within quadrants and quadrants within were fitted as random effects. To compare responses of each time interval during exposures, we ran a GLMM analysis accounting for data dependency of the repeated measures at five different times, that is, 1, 4, 8, 12, and 24 h after exposure. The random factor was formulated as the time interval nested within the ant colony and the ant colony with the exposure.

We determined which fixed factors significantly explained the variation of our response variable using a likelihood ratio test with the “anova” function, by comparing the model including the variable of interest with the same model without the variable of interest. When a fixed factor was significant and contained more than two groups, we computed contrast comparisons using post‐hoc tests, that is, Tukey test with Bonferroni adjusted *p*‐value, from the “emmeans” package. All statistics were carried out in R 4.0.3 (R Core Team, 2016) with the packages lme4 (Bates et al., 2015), emmeans (Lenth, 2016), DHARMa (Hartig, 2020), and glmmTMB (Brooks et al., 2017). Plots were created with the functions “plot,” “ggplot,” (Wilkinson, 2011) and “dplyr” package (Wickham et al., 2018).

## 3 RESULTS

### 3.1 Regulation and distinct responses toward fungi

**FIGURE 3:**
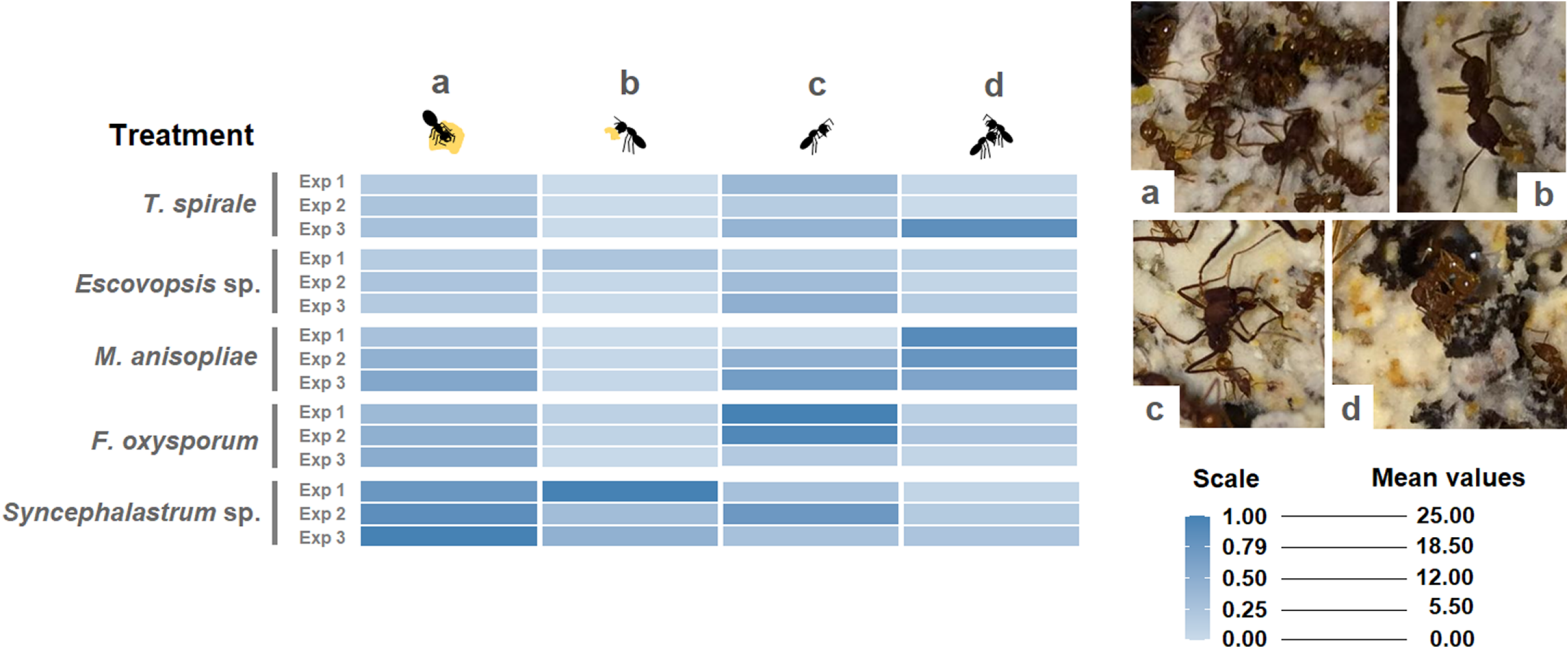
Distribution of the mean values for each cleaning behavior. Ants varied their responses, that is, (a) fungus grooming, (b) fungus weeding, (c) selfgrooming, and (d) allogrooming, according to the fungi treatment or control. The mean values are based on the sum of each counted behavior for each of the three exposures. The color scale is based on a rescaling of the numeric vector to an interval between 0 and 1 (“rescale” tool at the “dplyr” package), highlighting the minimum and maximum mean values. Fungus grooming was the most frequently registered behavior displayed by ants, while the others were less or not observed. See Supplementary S1 for the respective mean ± SE numbers. For the control distribution of each cleaning behavior, see Supplementary S3

**FIGURE 4:**
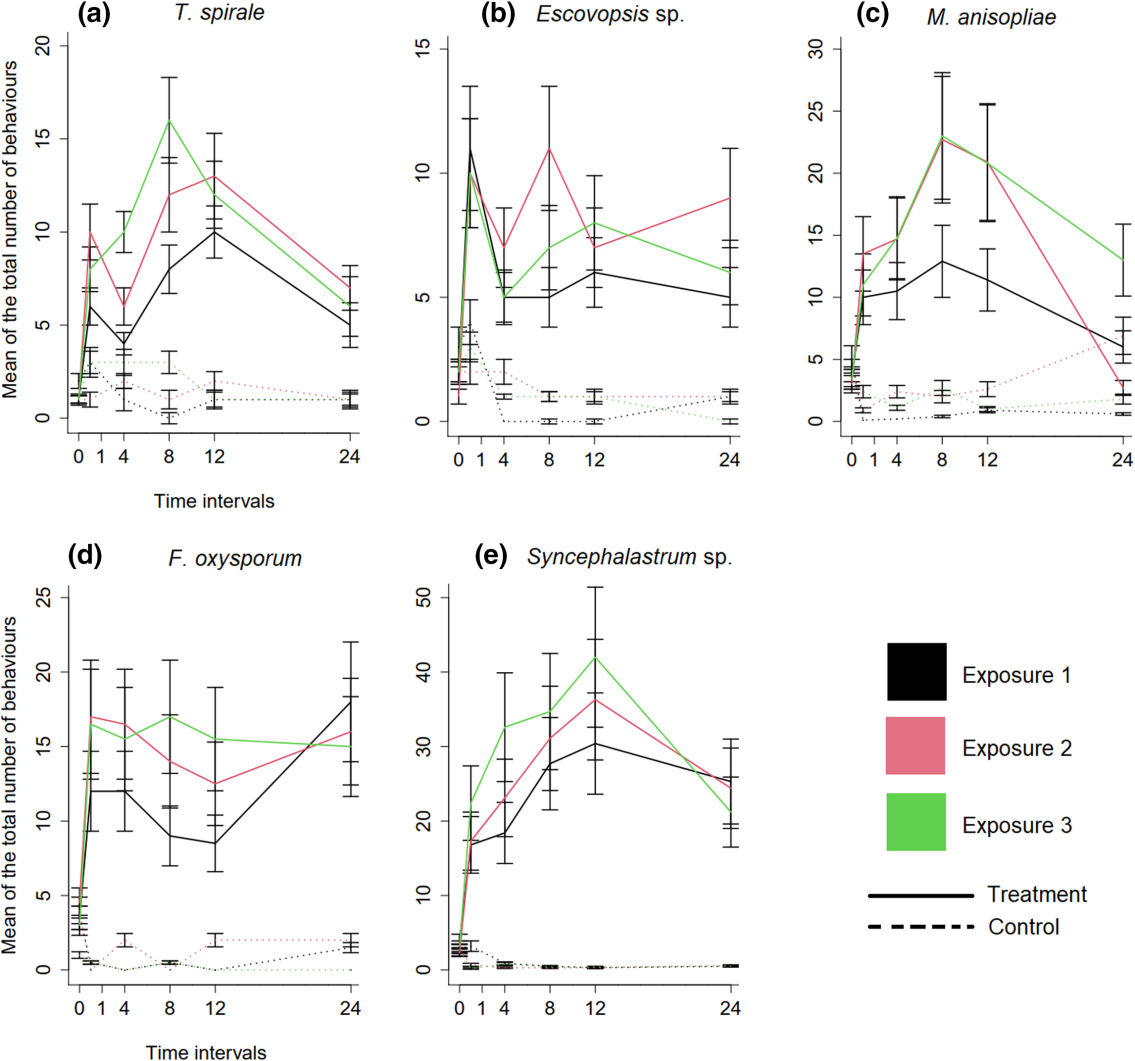
Response regulation over time for subsequent exposures. The graphs show the mean ± SE proportion of the total number counted of behaviors toward each fungal species (straight line) and its control (dashed line). Colonies showed variation on how they upregulated their responses, throughout the hours, to each fungus. (a) *Trichoderma spirale* and (b) *Escovopsis* sp., had the lowest increases, while (c) *Metarhizium anisopliae*, (d) *Fusarium oxysporum*, and (e) *Syncephalastrum* sp., elicited higher responses from the colonies. Different colors indicate specific exposures, that is, black is the first, red is the second, and green is the third exposure. Overall, the ants maintained similar patterns of their responses within the exposures, as different time points were not significantly different from each other (*post‐hoc* at *α* = .05). Controls showed an increase in the first hour followed by decreases and stabilization

One hour after inoculation, workers quickly detected conidia suspensions or the sham solution, and moved into the exposed area while increasing the number of hygienic behaviors, predominantly fungus grooming (Figure 3). Control treatments decreased and stabilized their responses within 1–4 h, while ants in fungal treatments showed a trend to continue displaying sanitary activity for 12 h in all exposures (Figure 4). There was no significant difference within time intervals, between exposures, regardless the treatment (Tukey's Test = *p* > .05 for all comparisons).

**FIGURE 5:**
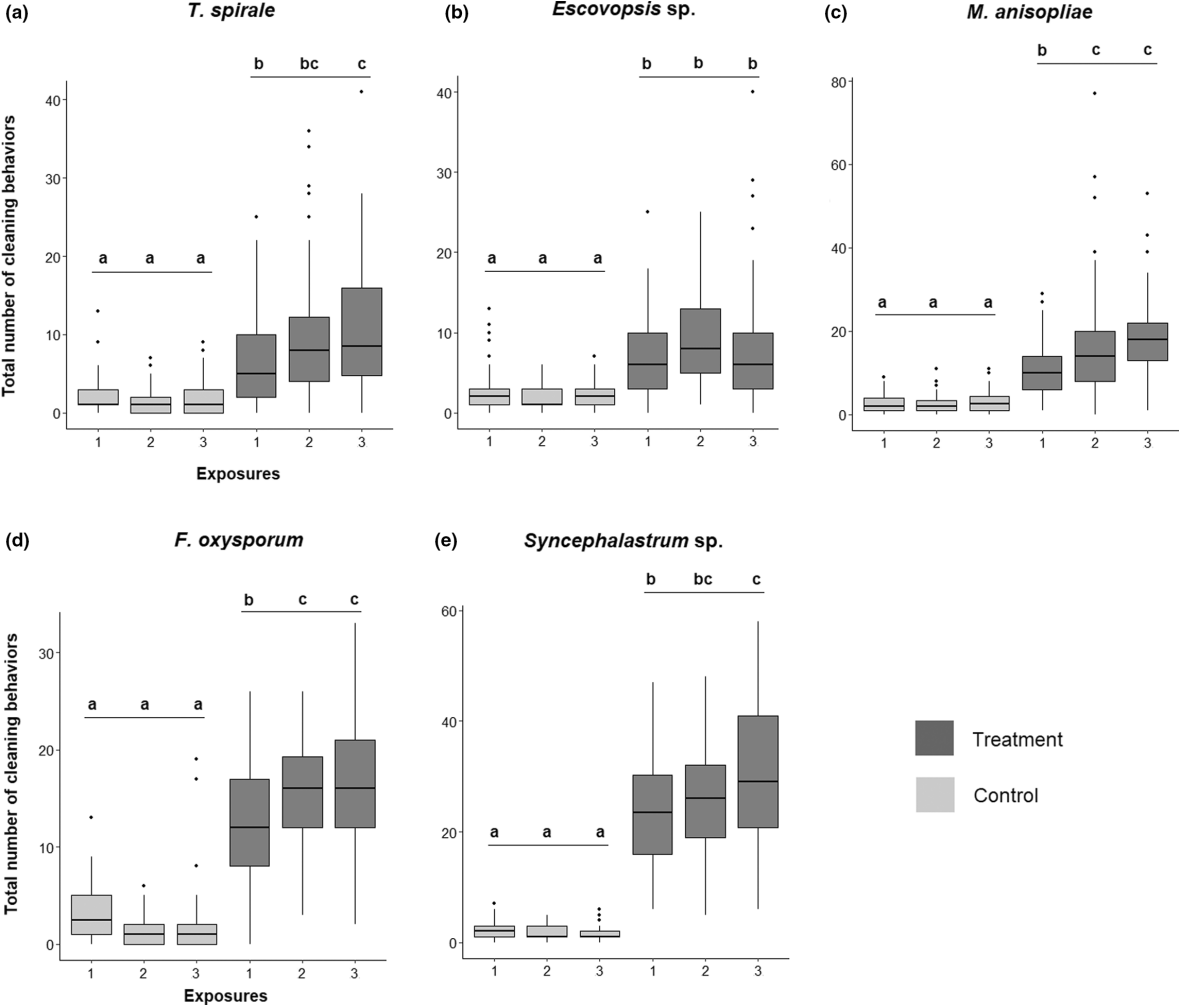
Distinct regulation of sanitization responses toward foreign fungi and their respective controls. The total number of counted cleaning responses for each treatment (median and quartiles 1 and 3), equals the sum of all responses given for each exposure. Fungus treatments incited a substantial increase of worker responses between first and third exposures. Fungus garden treated with (a) *Trichoderma spirale*, (c) *Metarhizium anisopliae*, and (e) *Syncephalastrum* sp., elicited increased reactions from workers between the first and third exposures. (d) *Fusarium oxysporum* showed significant increase between the first and third exposures, as well between the first and second one. (b) *Escovopsis* sp. did not elicit increased actions throughout exposures. Ant workers responded significantly lower to the sham solution in all cases regardless of exposures. Different letters in plots indicate statistical differences (*post‐hoc* at *α* = .05). Black points are outliers. For the median and quartiles of each behavior applied in treatments and exposures, see Supplementary S1

The total hygienic responses applied by ants were different when exposed to fungi in comparison with their respective control (*X* ^2^ = 402.2, *df* = 5, *p* < .001), regardless exposures (*T. spirale*: *X* ^2^ = 24.67, *df* = 1, *p* < .001; *Escovopsis* sp.: *X* ^2^ = 21.75, *df* = 1, *p* < .001; *M. anisopliae*: *X* ^2^ = 41.64, *df* = 1, *p* < .001; *Syncephalastrum* sp.: *X* ^2^ = 60.77, *df* = 1, *p* < .001; *F. oxysporum*: *X* ^2^ = 43.19, *df* = 1, *p* < .001; Figure 5). Also, we found that the hygienic effect differed between the inoculated fungal species (*X* ^2^ = 120.37, *df* = 12, *p* < .001; Figure 5). While *Escovopsis* sp. and *T. spirale* did not differ from each other (Tukey's Test = *p* > .05; Figure 5a,b), they elicited lower amounts of responses in comparison with other fungi (Tukey's Test = *p* < .001). The total number of behaviors between *F. oxysporum* and *M. anisopliae* were significantly different (Tukey's Test = *p* < .005), with higher responses seen for *F. oxysporum* (Tukey's Test = *p* < .005), except at the third exposure (Tukey's Test, *p* > .05; Figure 5d). The total number of hygienic responses toward *Syncephalastrum* sp. were higher in comparison with all other fungal species (Tukey's Test = *p* < .001; Figure 5e). Overall, the different fungi can be ordered from higher to low sanitization: *Syncephalastrum* sp. > *F. oxysporum* > *M. anisopliae* > *Escovopsis* sp. = *T. spirale*.

The hygienic strategy applied also varied according to the fungal species. Fungus grooming was the most common hygienic response expressed against all fungi (*X* ^2^ = 663.64, *df* = 4, *p* < .001; Figure 3, Supplementary S1). Selfgrooming was the second most common strategy applied (*X* ^2^ = 2428.9, *df* = 4, *p* < .001), with the highest numbers toward *F. oxysporum* and *M. anisopliae* (Figure 3, Supplementary S1). On the contrary, fungus weeding was less frequent in comparison with other behaviors (*X* ^2^ = 82.385, *df* = 4, *p* > .05), recorded at the first exposure for colonies treated with *Escovopsis* sp. and in colonies treated with *Syncephalastrum* sp. for all exposures (Supplementary S1 and S2). Allogrooming was observed in less quantity in all treatments (*X* ^2^ = 46.847, *df* = 4, *p* < .001), although substantially applied toward *M. anisopliae* in all exposures (Figure 3). In the control, allo‐, self‐, fungus grooming, and weeding were less common (Supplementary S1).

### 3.2 Responses under sequential exposures

There was a significant effect for exposures within treatment (*Escovopsis* sp.: *X* ^2^ = 10.186, *df* = 4, *p* < .005; *F. oxysporum*: *X* ^2^ = 37.646, *df* = 4, *p* < .001; *M. anisopliae*: *X* ^2^ = 31.719, *df* = 4, *p* < .001; *Syncephalastrum* sp.: *X* ^2^ = 23.001, *df* = 4, *p* < .001; *T. spirale*: *X* ^2^ = 10.364, *df* *df* = 4, *p* < .005). In general, the repetition of fungus exposures resulted in increased rates of the total hygienic responses (Tukey's Test = *p* < .05, see comparisons between exposures in Figure 5 and Table 1); however, this was mostly influenced by fungus grooming (Figure 3; Supplementary S1). Responses to *T. spirale*, *M. anisopliae*, *F. oxysporum*, and *Syncephalastrum* sp. increased significantly between first and third exposures (Tukey's Test = *p* < .05; Table 1). There was also significant increase between the first and second exposure toward *F. oxysporum* and *M. anisopliae* (Tukey's Test = *p* < .05; Table 1). In contrast, for *Escovopsis* sp., the responses were not statistically distinct throughout exposures (Tukey's Test = *p* > .05; Table 1). Sanitizations in the controls were not significantly distinct throughout exposures and did not show patterns or indications that suggested an increase in hygiene (Tukey's Test = *p* > .05, see Figure 5; Table 1).

**TABLE 1:** *Post‐hoc* test comparing exposures of each fungus. The consecutive encounters with each fungus resulted in a different number of responses from the colonies at the first and/or third exposures (Tukey's test, *α* = .05). In general, these differences showed an increase in the total number of hygienical behaviors toward all fungi, but *Escovopsis* sp. (Figure 3). Ants significantly increased their responses from Exp 1 to Exp 3 when inoculated with *Fusarium oxysporum, Metarhizium anisopliae, Syncephalastrum* sp., and *Trichoderma spirale*

Treatments	Comparison	*Z*‐score	*p*
*Escovopsis* sp.	Exp 1 × Exp 2	−2.250	.2065
	Exp 2 × Exp 3	1.951	.3722
	Exp 3 × Exp 1	−0.326	.9995
*F. oxysporum*	Exp 1 × Exp 2	−3.931	<.005
	Exp 2 × Exp 3	−1.012	.9140
	Exp 3 × Exp 1	−4.929	<.001
*M. anisopliae*	Exp 1 × Exp 2	−4.047	<.005
	Exp 2 × Exp 3	−2.159	.2588
	Exp 3 × Exp 1	−6.175	<.001
*Syncephalastrum* sp.	Exp 1 × Exp 2	−2.458	.1385
	Exp 2 × Exp 3	−2.583	.1029
	Exp 3 × Exp 1	−5.023	<.001
*T. spirale*	Exp 1 × Exp 2	−2.250	.2166
	Exp 2 × Exp 3	−0.724	.9790
	Exp 3 × Exp 1	−2.963	<.005

## 4 DISCUSSION

### 4.1 Responses toward distinct fungi

In the last decades, it has been argued that leaf‐cutting ants show specific removal and cleaning responses against pathogenic fungi (Currie & Stuart, 2001; Mighell & Van Bael, 2016; Tranter et al., 2015). Indeed, after analyzing 15 h of video footage, we show that ant workers change the total amount of sanitary care, predominantly fungus grooming, and regulated the intensity of specific behaviors depending on the fungus the colony is exposed to. As previously reported (Bonadies et al., 2019; Mighell & Van Bael, 2016), our results also show the ability of leaf‐cutting ant colonies to distinguish conidia from different fungal species. More research is necessary to evaluate whether these differences and enhancement of sanitization result in fast and efficient removal of fungi.

Leaf‐cutting ants may vary their detection threshold and cleaning responses to fungi based on their antagonistic interaction (Currie & Stuart, 2001; Goes et al., 2020; Mighell & Van Bael, 2016) and/or lifelong pressures (Boomsma et al., 2005). *Escovopsis* is generally considered a virulent mycoparasite to the fungus garden (Currie, 2001); however, sanitization toward it was low and similar to that seen for *T. spirale* (Figure 5). Even though *Escovopsis* sp. was removed more than *T. spirale* at the second exposure, including fungus weeding (Supplementary S1), the total amount of sanitization was still lower in comparison with the other antagonistic fungi. This suggests that *Escovopsis* sp., or at least the specific strain used in this study (LESF 021), may not be as virulent and pathogenic to the fungus garden as previously thought (Currie, 2001; Currie & Stuart, 2001). An alternative scenario would be the presence of other *Escovopsis* spp. strains in the colonies which may have affected virulence of LESF 021. Interactions among *Escovopsis* strains cohabiting the same host may result in competition, inhibiting each other in the system (Christopher et al., 2021b). Nevertheless, some associations are dynamic and can shift on the parasitism–mutualism continuum in response to environmental changes and/or the host susceptibility (Brown et al., 2012; Jiménez‐Gómez et al., 2021; Leung & Poulin, 2008; Mendonça et al., 2021). The capacity of *Escovopsis* to cause disease at the system‐level, and therefore trigger higher sanitization, may depend on the health and susceptibility condition of the superorganism, that is, the ants, the fungal cultivar, and the symbiotic community (Jiménez‐Gómez et al., 2021; Mendonça et al., 2021). Even so, we cannot exclude the chance that ants applied other prophylactic behaviors that were not measured in our study, such as metapleural gland grooming and the further discharge of infrabuccal pellets (Fernández‐Marín et al., 2013, 2015; Yek et al., 2012). One of the components found in these glands, the phenylacetic acid, is efficient against the germination of *Escovopsis* strains and even of *M. brunneum* (Fernández‐Marín et al., 2015). Because grooming behaviors may be linked to the use of these glands, it is likely that ants applied this chemical defensive strategy.

In contrast to *Escovopsis* sp. and *T. spirale*, ants intensively sanitized against *Syncephalastrum* sp. (Figure 3). Infection to this fungus has been shown to destabilize *A. sexdens* queen‐less colonies with workers stopping foraging activities, increases in ant mortality, and disposal of the fungus garden (Barcoto et al., 2017). It is postulated that *Syncephalastrum* sp. is a generalist pathogen that might germinate quickly in the fungus garden (Barcoto et al., 2017). Thus, to avoid sudden fungus garden deterioration, fungus weeding was greatly applied one hour after its inoculation (Supplementary S2). Although we noticed indirect occurrences of fungus weeding by the disposed fungus garden (Supplementary S2), we cannot evaluate how significant it was throughout exposures and to other fungi than *Syncephalastrum* sp. As this behavior generally took longer than the length of our recordings, it would have been harder to identify properly.

Because leaf‐cutting ants might frequently be exposed to *M. anisopliae*, and because of its entomopathogenic nature, self‐ and allogrooming are expected sanitization behaviors to prevent mortalities (Lacerda et al., 2010; Lopez & Orduz, 2003; Morelos‐Juárez et al., 2010). Indeed, physical removal of contaminants from workers and nestmates was present (Figure 3). Nevertheless, we also observed intense fungus grooming applied against this entomopathogen. Such responses from ants would not be a surprise, once the location/context where the contaminant is found may be a predictor of the strategy chosen (Yek et al., 2013). Reasonably, even not being a direct threat to the fungal cultivar, ants would benefit from removing *M. anisopliae* conidia found on it or in any other part of the colony (Tranter et al., 2015), preventing further general contamination.

Lastly, although *F. oxysporum* is commonly isolated from leaf‐cutting ant nests (Rodrigues et al., 2008), no antagonistic relationship has been reported so far. *F. oxysporum* strains vary in ecological role, ranging from nonpathogenic endophytes colonizing plant roots (Benhamou et al., 2002) to causing disease (Ploetz et al., Ploetz, 2006; Dita et al., 2018). The leaf‐cutting ant *Atta laevigata* rejects leaves that contain endophytic *Fusarium* spp. (Rocha et al., 2014) suggesting that some strains act to protect plants from herbivory, potentially harming the fungus crop, similar to the Trojan‐horse hypothesis suggested for *Trichoderma* (Rocha et al., 2017). This could explain the intensive responses seen toward *F. oxyporum*, although more research is required to better understand its impact on leaf‐cutting ant colonies.

Some ambrosia beetles, fungus‐growing termites, and leaf‐cutting ants of the genus *Acromyrmex* detect volatile organic compounds (VOCs) or other chemical blends from their symbionts (Davis et al., 2013; Huler et al., 2011; Katariya et al., 2017; Zhang et al., 2007). They can use chemical profiles to discriminate native from non‐native microbes (Christopher et al., 2021a; Richard et al., 2007; Zhang et al., 2007), to collect cultivar conidia from the environment (Biedermann & Kaltenpoth, 2014), and even to influence host behavior (Davis et al., 2013). It is, therefore, plausible that the recognition of chemical cues from non‐native microbes influences the intensity of hygienical responses (Goes et al., 2020; Katariya et al., 2017; Yanagawa et al., 2011). Besides the ecological role hypothesis, differences in the chemical profiles of the fungal conidia and that of the colony could trigger distinct reactions by ants (Goes et al., 2020). In addition, termites (Katariya et al., 2017; Yanagawa et al., 2011) and honeybees (Swanson et al., 2009) are able to discriminate foreign species and sanitize accordingly. As previously suggested (Christopher et al., 2021a), it is necessary to investigate whether different phenotypic traits of conidia, for example, odor, growth rate, morphology, or size, can modulate species‐specific actions at the colony and individual level in attine ants.

### 4.2 Responses after successive fungi exposures

In addition to the ability to recognize alien fungi and respond differently to them, we investigated how *A. sexdens* deals with repeated exposure to the same fungus. We show that the ants tended to increase sanitization after three exposures to the same fungus (Figures 4 and 5). Except for *Escovopsis* sp., overall responses increased from the first to the third exposure (Figure 5), suggesting that the system sensitized through previous encounters. However, the question of how and why this increase is established still remains. Social insects can learn through experiences and enhance the performance of their tasks (Giurfa, 2007; Leadbeater & Chittka, 2007) and by combining multiple individual experiences, they can improve group actions (Sasaki & Pratt, 2018). As a result, group experience‐modulated actions influence (1) the flexibility in colonies to deal with recurrent pathogens, (2) reaction time, and/or (3) the intensity of cleaning tasks, increasing the efficiency of contaminant removal (Konrad et al., 2018; Reber et al., 2011; Walker & Hughes, 2009; Westhus et al., 2014). In addition, contact with sick individuals is reduced and avoided in relation to the infection history (Konrad et al., 2018). Therefore, we suggest further studies investigating the correlation between collective experience and fast and efficient removal of contaminants in social insect systems.

The response of *A. sexdens* increased even after a two‐week interval, suggesting that colonies may have retained information regarding the threat level of the specific fungus and increased the efficiency of their responses throughout exposures (Pull & McMahon, 2020). Nevertheless, we cannot simply conclude that an association exists between increasing in responses and the retention of information by ants. To test for such association, it would be necessary to use an unspecific second or third exposure, with a sham solution or another fungus, that is, heterologous exposure (Sadd & Schmid‐Hempel, 2006). Hypothetically, if the ants retain information from past exposures to the same pathogen, we expect them to increase their responses only to the pathogen, and not be affected by an unspecific exposure. Otherwise, it would indicate that other clues from the infection are sensitizing the ants or the fungus garden. Therefore, further study is required to investigate (1) which information is retained by the system in hazardous experiences, (2) how it may affect or not their strategies, and (3) whether past experiences are related to defensive plasticity, that is, immunological specificity and enhanced responses.

## 5 CONCLUSIONS

Leaf‐cutting ants encounter a myriad of microorganisms that potentially outcompete their beneficial symbionts. For this reason, partner screening and discrimination is a key aspect to the maintenance of this mutualistic association. Our study shows that colonies of the leaf‐cutting ant *A. sexdens* can discriminate and respond distinctively to five fungal species. Our results corroborate with previous studies that indicate species‐specific removal and adjustment of defensive behaviors in attine ants (Christopher et al., 2021a; Currie & Stuart, 2001; Fernández‐Marín et al., 2013; Mighell & Van Bael, 2016; Tranter et al., 2015; Yek et al., 2012). In addition, this is the first study that shows plasticity in this ant species through repeated exposures to the same fungus. Increased responses were seen after one and/or two previous exposures, indicating that *A. sexdens* colonies change their response due to their infection history. Whether such increased response contributes to faster and more efficient removal of the contaminants (Westhus et al., 2014), remains to be answered. Biological control of *A. sexdens* found in agricultural crops may benefit from our findings. As seen in this study, species‐specific and plasticity to defend the symbiosis in future infections may hamper repeated applications of a single microbial pathogen. Perhaps, heterologous exposures by exchanging strains during repeated applications would avoid ants to be sensitized to a particular pathogen. Lastly, because of our experimental design, we cannot be sure whether such discrimination and plasticity from exposure to exposure were due to the ants, the fungus garden or a combination from both. Elucidating such aspects will improve our understanding of how both parties maintain the stability of the leaf‐cutting ant–fungus mutualism.

## AUTHOR CONTRIBUTIONS

**Aryel C. Goes:** Conceptualization (lead); data curation (equal); formal analysis (equal); funding acquisition (equal); investigation (lead); methodology (lead); software (equal); validation (equal); visualization (equal); writing – original draft (lead); writing – review and editing (supporting). **Pepijn Kooij:** Data curation (equal); formal analysis (equal); funding acquisition (equal); investigation (equal); resources (supporting); software (supporting); supervision (supporting); validation (equal); visualization (equal); writing – original draft (supporting); writing – review and editing (supporting). **Laurence Culot:** Conceptualization (supporting); data curation (equal); formal analysis (equal); investigation (supporting); methodology (equal); software (equal); supervision (supporting); validation (supporting); visualization (supporting); writing – original draft (supporting); writing – review and editing (supporting). **Odair Bueno:** Conceptualization (equal); formal analysis (supporting); investigation (supporting); methodology (equal); project administration (supporting); resources (equal); supervision (equal); validation (equal); visualization (equal); writing – original draft (supporting); writing – review and editing (supporting). **Andre Rodrigues:** Conceptualization (equal); data curation (equal); funding acquisition (lead); investigation (equal); methodology (equal); project administration (lead); resources (lead); supervision (lead); validation (lead); visualization (equal); writing – original draft (supporting); writing – review and editing (supporting).

## CONFLICT OF INTEREST

The authors declare that there is no conflict of interest.

## FUNDING INFORMATION

AR received funding from Fundação de Amparo à Pesquisa do Estado de São Paulo (FAPESP), grants #2019/03746–0 and #2012/25299–6; and from Conselho Nacional de Desenvolvimento Científico e Tecnológico (CNPq), grant #305269/2018–6. ACG received a scholarship from FAPESP, #2019/03087–6. The contribution to this paper by PK was possible thanks to the scholarship granted from the Coordenação de Aperfeiçoamento de Pessoal de Nível Superior (CAPES), in the scope of the Program CAPES‐PrInt, process number 88887.310463/2018–00, Mobility numbers #88887.468939/2019–00 and #88887.571230/2020–00.

## Supporting information


**Supplementary S1** Mean number ± standard error of the mean (SE) of ants displaying the different hygienic behaviors in relation to the treatment and exposure of each fungi species. Means are based on count data. Generally, the colony applied fungus grooming to remove the conidia or sham solution. Self‐ and allogrooming were common toward *Trichoderma spirale* and *Metarhizium anisopliae*. Weeding was less present for the majority of fungi and absent for *T. spirale*. We subtracted the number of behaviors accounted at “one hour before inoculation” from each of the time points to set the baseline behaviors of ants. EXP 1, EXP 2, and EXP 3 identify exposures 1, 2, and 3, respectively.
**Supplementary S2** Evidence of fungus weeding behavior in colonies of *Atta sexdens*. Fungus weeding is the removal of large infected pieces of the fungus garden, and their disposal at the dump chamber. (A) Dump chamber of a colony treated with conidia of *Escovopsis* sp., 24 h postinoculation, showing debris of the fungus garden. (B) Workers carrying fragments of the fungus garden treated with *Syncephalastrum* sp. (arrows), 1 h postinoculation. (C) Dump chamber of a colony treated with *Metarhizium anisopliae*, showing rejected pieces of the fungus garden, and (D) the waste dump contained many contaminated pieces of fungus garden with *Syncephalastrum* sp. 24 h post‐inoculation. Images were taken during and after the first exposure.
**Supplementary S3** Distribution of the mean total number of each cleaning behavior for the control colonies that received the sham solution. Ants varied the number and type of responses: (a) fungus grooming, (b) fungus weeding, (c) selfgrooming, and (d) allogrooming. The mean values are based on the sum of each counted behavior for each of the three exposures. The color scale is based on a rescaling of the numeric vector to an interval between 0 and 1, highlighting the minimum and maximum mean values. Fungus grooming and selfgrooming was the most common and registered behavior by ants, while the others were less or not observed. Figures on the top right corner represent the four cleaning behaviors.

## Data Availability

The dataset analyzed and used to produce Figures 3, 5, and Table 1, is publicly available in figshare repository (https://doi.org/10.6084/m9.figshare.14330462). Data used to produce Figure 4 and Supplementary S1 are also avaliable in figshare repository (https://doi.org/10.6084/m9.figshare.14330468). All video recordings that resulted in the raw data are not publicly available due to the total file size, however, the material is stored on ACG's personal drive for review. Specific recordings and the R scripts are available from the corresponding author upon request.
