# Supplementary material for: Distinct and enhanced hygienic responses of a leaf‐cutting ant toward repeated fungi exposures
Source: Ecol Evol. 2022 Jul 17;12(7):e9112. doi: 10.1002/ece3.9112 (PMC9288931; doi:10.1002/ece3.9112)
Supplement: Supplementary file 1 — Supplementary S1 Mean number ± standard error of the mean (SE) of ants displaying the different hygienic behaviors in relation to the treatment and exposure of each fungi species. Means are based on count data. Generally, the colony applied fungus grooming to remove the conidia or sham solution. Self‐ and allogrooming were common toward Trichoderma spirale and Metarhizium anisopliae. Weeding was less present for the majority of fungi and absent for T. spirale. We subtracted the number of behaviors accounted at “one hour before inoculation” from each of the time points to set the baseline behaviors of ants. EXP 1, EXP 2, and EXP 3 identify exposures 1, 2, and 3, respectively. Supplementary S2 Evidence of fungus weeding behavior in colonies of Atta sexdens. Fungus weeding is the removal of large infected pieces of the fungus garden, and their disposal at the dump chamber. (A) Dump chamber of a colony treated with conidia of Escovopsis sp., 24 h postinoculation, showing debris of the fungus garden. (B) Workers carrying fragments of the fungus garden treated with Syncephalastrum sp. (arrows), 1 h postinoculation. (C) Dump chamber of a colony treated with Metarhizium anisopliae, showing rejected pieces of the fungus garden, and (D) the waste dump contained many contaminated pieces of fungus garden with Syncephalastrum sp. 24 h post‐inoculation. Images were taken during and after the first exposure. Supplementary S3 Distribution of the mean total number of each cleaning behavior for the control colonies that received the sham solution. Ants varied the number and type of responses: (a) fungus grooming, (b) fungus weeding, (c) selfgrooming, and (d) allogrooming. The mean values are based on the sum of each counted behavior for each of the three exposures. The color scale is based on a rescaling of the numeric vector to an interval between 0 and 1, highlighting the minimum and maximum mean values. Fungus grooming and selfgrooming was the most common and registered behavior b [file ECE3-12-e9112-s001.docx]

SUPPLEMENTARY MATERIAL

Differential and enhanced hygienic responses of a leaf-cutting ant due to repeated fungi exposures

Aryel C. Goes^1^, Pepijn W. Kooij^1^, Laurence Culot^2^, Odair C. Bueno^1^, Andre Rodrigues^1*^

^1^Department of General and Applied Biology, São Paulo State University (UNESP), Rio Claro, Brazil.

^2^Department of Biodiversity, São Paulo State University (UNESP), Rio Claro, Brazil.


**Supplementary S1** Mean number ± standard error of the mean (SE) of ants displaying the different hygienic behaviors in relation to the treatment and exposure of each fungi species. Means are based on count data. Generally, the colony applied fungus grooming to remove the conidia or sham solution. Self- and allogrooming were common towards *Trichoderma spirale* and *Metarhizium anisopliae.* Weeding was less present for the majority of fungi and absent for *T. spirale*. We subtracted the number of behaviors accounted at “one hour before inoculation” from each of the time points to set the baseline behaviors of ants. EXP 1, EXP 2, and EXP 3 identify exposures 1, 2, and 3, respectively.

| **Treatments** |  |  | **MEAN ± SE** | | | |
| --- | --- | --- | --- | --- | --- | --- |
|  |  |  | **Fungus Grooming** | **Fungus Weeding** | **Selfgrooming** | **Allogrooming** |
| *T. spirale* | EXP 1 | Treatment | 6.78±0.54 | 0.00 | 0.17±0.05 | 0.03±0.03 |
|  |  | Control | 2.80±0.28 | 0.00 | 0.24±0.03 | 0.11±0.03 |
|  | EXP 2 | Treatment | 9.00±0.54 | 0.00 | 0.08±0.06 | 0.01±0.03 |
|  |  | Control | 1.85±0.19 | 0.00 | 0.25±0.04 | 0.07±0.03 |
|  | EXP 3 | Treatment | 9.81±0.76 | 0.00 | 0.19±0.07 | 0.39±0.08 |
|  |  | Control | 2.67±0.27 | 0.00 | 0.18±0.03 | 0.09±0.03 |
| *Escovopsis* sp*.* | EXP 1 | Treatment | 6.37±0.55 | 0.11±0.03 | 0.07±0.05 | 0.06±0.02 |
|  |  | Control | 3.61±0.30 | 0.03±0.05 | 0.24±0.03 | 0.03±0.01 |
|  | EXP 2 | Treatment | 8.83±0.56 | 0.02±0.01 | 0.15±0.04 | 0.04±0.04 |
|  |  | Control | 3.18±0.20 | 0.00 | 0.16±0.03 | 0.15±0.04 |
|  | EXP 3 | Treatment | 6.92±0.67 | 0.00 | 0.21±0.05 | 0.08±0.03 |
|  |  | Control | 2.82±0.17 | 0.00 | 0.13±0.03 | 0.09±0.04 |
| *M. anisopliae* | EXP 1 | Treatment | 9.73±0.67 | 0.00±0.01 | 0.01±0.09 | 0.41±0.09 |
|  |  | Control | 8.66±0.88 | 0.00 | 0.25±0.04 | 0.29±0.05 |
|  | EXP 2 | Treatment | 14.87±1.22 | 0.02±0.02 | 0.21±0.06 | 0.36±0.07 |
|  |  | Control | 6.57±0.54 | 0.00 | 0.22±0.06 | 0.46±0.08 |
|  | EXP 3 | Treatment | 17.92±0.90 | 0.02±0.01 | 0.30±0.09 | 0.28±0.07 |
|  |  | Control | 4.63±0.25 | 0.00 | 0.18±0.04 | 0.34±0.06 |
| *F. oxysporum* | EXP 1 | Treatment | 12.10±0.77 | 0.05±0.03 | 0.43±0.08 | 0.07±0.03 |
|  |  | Control | 3.33±0.28 | 0.00 | 0.21±0.05 | 0.04±0.02 |
|  | EXP 2 | Treatment | 15.27±0.52 | 0.04±0.02 | 0.40±0.07 | 0.12±0.04 |
|  |  | Control | 2.31±0.21 | 0.00 | 0.17±0.04 | 0.07±0.03 |
|  | EXP 3 | Treatment | 16.34±0.64 | 0.01±0.01 | 0.09±0.08 | 0.05±0.02 |
|  |  | Control | 1.99±0.62 | 0.00 | 0.18±0.04 | 0.04±0.02 |
| *Syncephalastrum* sp. | EXP 1 | Treatment | 23.19±0.94 | 0.46±0.12 | 0.13±0.06 | 0.04±0.04 |
|  |  | Control | 3.35±0.23 | 0.02±0.02 | 0.21±0.04 | 0.04±0.02 |
|  | EXP 2 | Treatment | 25.97±1.05 | 0.15±0.06 | 0.32±0.06 | 0.9±0.06 |
|  |  | Control | 2.27±0.15 | 0.00 | 0.9±0.03 | 0.04±0.02 |
|  | EXP 3 | Treatment | 30.11±1.35 | 0.21±0.05 | 0.13±0.10 | 0.12±0.04 |
|  |  | Control | 2.00±0.15 | 0.01±0.01 | 0.14±0.04 | 0.04±0.02 |

**Supplementary S2** Evidence of fungus weeding behavior in colonies of *Atta sexdens*. Fungus weeding is the removal of large infected pieces of the fungus garden, and their disposal at the dump chamber. (A) Dump chamber of a colony treated with conidia of *Escovopsis* sp., 24 h post-inoculation, showing debris of the fungus garden. (B) Workers carrying fragments of the fungus garden treated with *Syncephalastrum* sp. (arrows), 1 h post-inoculation. (C) Dump chamber of a colony treated with *Metarhizium anisopliae*, showing rejected pieces of the fungus garden, and (D) the waste dump contained many contaminated pieces of fungus garden with *Syncephalastrum* sp. 24 h post-inoculation. Images were taken during and after the first exposure.


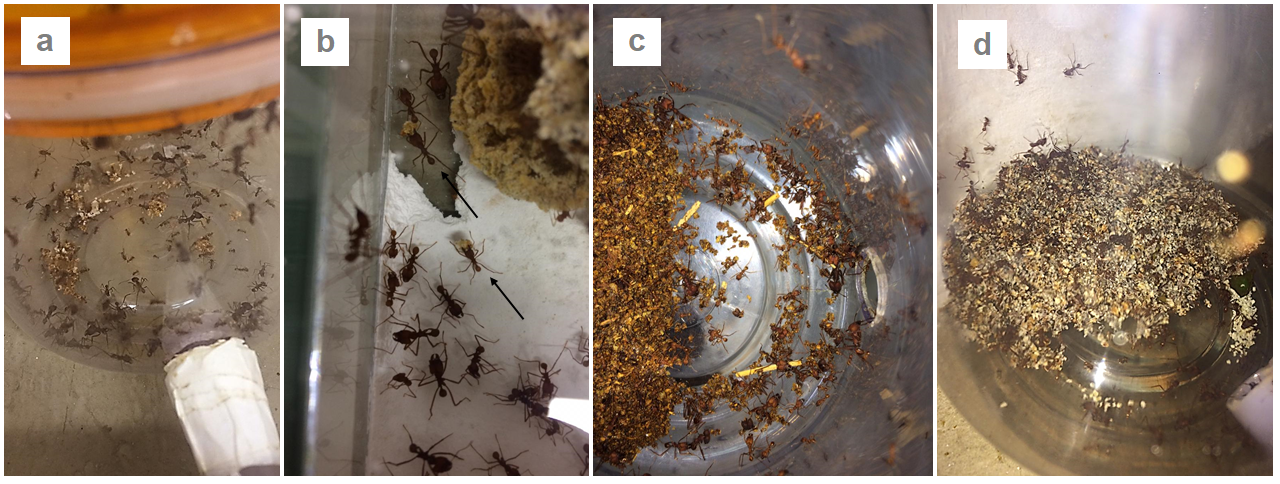


**Supplementary S3** Distribution of the mean total number of each cleaning behavior for the control colonies that received the sham solution. Ants varied the number and type of responses, i.e., (a) fungus grooming, (b) fungus weeding, (c) selfgrooming, and (d) allogrooming, towards. The mean values are based on the sum of each counted behavior for each of the three exposures. The colour scale is based on a rescaling of the numeric vector to an interval between 0 and 1, highlighting the minimum and maximum mean values. Fungus grooming and selfgrooming was the most common and registered behavior by ants, while the others were less or not observed. Figures on the top right corner represent the four cleaning behaviors.

**
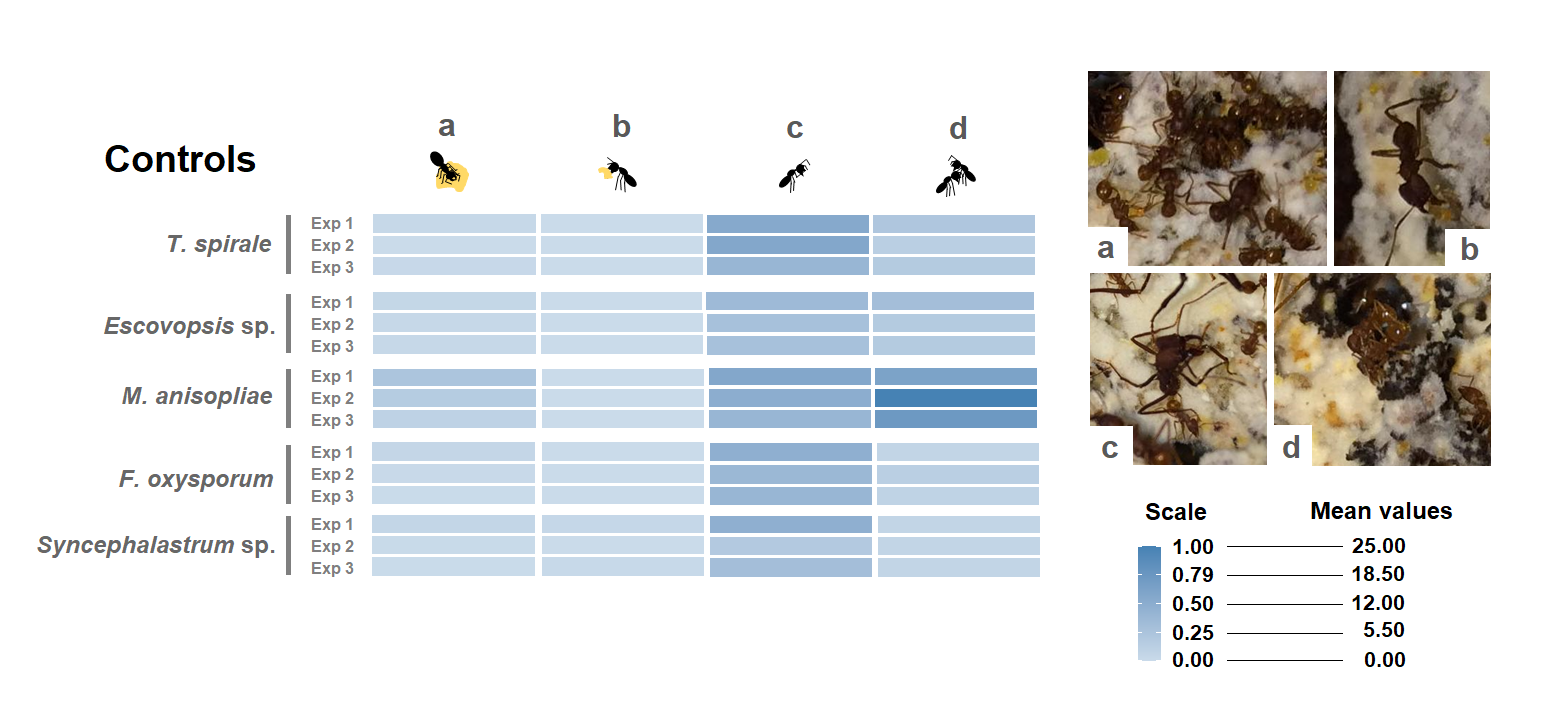
**
